# Association of estimated pulse wave velocity with all-cause mortality and cardiovascular mortality in obstructive sleep apnea patients: results from NHANES

**DOI:** 10.3389/fcvm.2025.1571610

**Published:** 2025-06-12

**Authors:** Huizhong Lin, Huiyun Zheng, Tao Lin, Lianglong Chen

**Affiliations:** ^1^ Department of Cardiology, Fujian Medical Center for Cardiovascular Diseases, Fujian Institute of Coronary Heart Disease, Fujian Medical University Union Hospital, Fuzhou, China; ^2^ Department of Epidemiology, School of Public Health, Fujian Medical University, Fuzhou, China

**Keywords:** estimated pulse wave velocity, obstructive sleep apnea, arterial stiffness, NHANES, all-cause mortality, cardiovascular mortality

## Abstract

**Background:**

This study aims to investigate the relationship between estimated pulse wave velocity (ePWV) and all-cause mortality (ACM) and cardiovascular mortality (CVM) in patients with obstructive sleep apnea (OSA).

**Method and results:**

A cohort study was conducted using data from the NHANES database (2005–2008, 2015–2018), focusing on adults with OSA. ePWV was calculated based on age and mean blood pressure. A weighted Cox regression model analyzed the association of ePWV with ACM and CVM, while restricted cubic spline (RCS) curves visualized this relationship. Kaplan–Meier (KM) survival curves assessed survival across different ePWV groups.The study involved 10,071 OSA patients with an average age of 48.46 years, with a follow-up period ending in December 2019 (average follow-up time: 102.10 ± 2.34 months). Results showed that increased ePWV correlated with higher ACM [hazard ratio [HR]: 1.25, 95% confidence interval [CI]: 1.20–1.31] and CVM (HR: 1.28, 95% CI: 1.21–1.36). RCS curves indicated stable mortality risks at ePWV ≤ 8.1 m/s, with rapid increases beyond this threshold. KM curves demonstrated poorer survival outcomes for OSA patients with elevated ePWV.

**Conclusion:**

Elevated ePWV levels are linked to increased ACM and CVM in OSA patients, suggesting that monitoring ePWV could help mitigate these risks and promote healthier longevity in this population.

## Introduction

Sleep apnea is a prevalent sleep disorder characterized by the recurrent cessation and initiation of breathing (apneas or hypopneas) during sleep (1, 2). The most common type of sleep-related breathing disorder is obstructive sleep apnea (OSA), with reports indicating that approximately one billion individuals worldwide are affected by OSA, and this number continues to escalate (3). The primary clinical hallmark of OSA is the repetitive collapse of the upper airway during sleep due to anatomical narrowing and increased pharyngeal compliance, leading to obstruction (4, 5). The most pronounced external impact is the loud, frequent, and bothersome snoring that occurs during sleep, which not only disrupts nocturnal rest and may even result in episodes of suffocation during sleep but also impairs daytime alertness (6, 7). Studies suggested that OSA may be a potential independent risk factor for sudden cardiac death (8). Besides, an increasing number of studies confirm that the risk of all-cause death was significantly increased in patients with moderate to severe or severe OSA (9, 10). Consequently, it is essential to pay attention to the health status of patients with OSA, strive to reduce the burden related to mortality, and ensure a better healthy lifespan.

Arterial stiffness (AS), also known as the loss of arterial elasticity, is a reliable indicator for assessing changes in arterial structure and function, and it is also an important cardiovascular risk factor (11, 12). AS is widely recognized as a significant predictor of cardiovascular events and mortality, including all-cause mortality (ACM) and cardiovascular mortality (CVM) (13, 14). Currently, carotid-femoral pulse wave velocity (cf-PWV) is a common method for assessing AS (15). However, the measurement of cf-PWV requires expensive specialized equipment and professional personnel (16). On the one hand, this leads to its limited use in clinical practice, and on the other hand, it restricts its widespread application in large-scale, large-sample population epidemiological studies (17). Researchers have proposed a new concept of estimating pulse wave velocity (ePWV). ePWV is a new indicator calculated based on mean blood pressure (MBP) and age (18). Previous studies have found that ePWV has good predictive value for stroke, myocardial infarction, CVM, and other outcomes (19, 20). Moreover, studies have shown a strong correlation between ePWV and measured cfPWV, suggesting that ePWV measurement can be used to monitor the severity of AS (21, 22).

Published studies have demonstrated a close association between OSA and AS (23–26), that OSA often promotes the development of AS. Patients with OSA often present with endothelial dysfunction, which contributes to the development of atherosclerosis (27). Additionally, the chronic intermittent hypoxia associated with OSA can activate signaling pathways that promote inflammation and vascular remodeling, leading to the progression of atherosclerotic plaques (28, 29). Tang et al. also found that compared to the general population, individuals with OSA have increased AS, which may rise significantly with the severity of OSA (30). The relationship between OSA and AS thus holds significant clinical implications, underscoring the need for OSA patients to be more vigilant about arteriosclerosis. Although several studies have investigated the relationship between ePWV and mortality in the general population, stroke patients, hypertensive population, and those with diabetes, to our knowledge, the association between ePWV and mortality among high-risk OSA patients remains uncharted (12, 17, 31, 32). Against this research backdrop, in the present study, we utilized the large multi-ethnic cohort data from the National Health and Nutrition Examination Survey (NHANES) to assess the relationship between AS, as indicated by ePWV, and ACM and CVM in patients with OSA.

## Methods

### Data source

The NHANES is a nationwide study conducted by the National Center for Health Statistics (NCHS) in the United States, focusing on the health and nutritional status of U.S. adult and pediatric populations. Using a complex multi-stage sampling design, the survey is conducted biennially to monitor the prevalence of diseases by examining the health and nutritional conditions of the aforementioned groups (33). Additionally, each participant provides the necessary written informed consent before starting investigations. All NHANES data is publicly available and can be freely downloaded through the following means: https://www.cdc.gov/nchs/nhanes/index.htm.

### Study design and population selection

**Figure 1:**
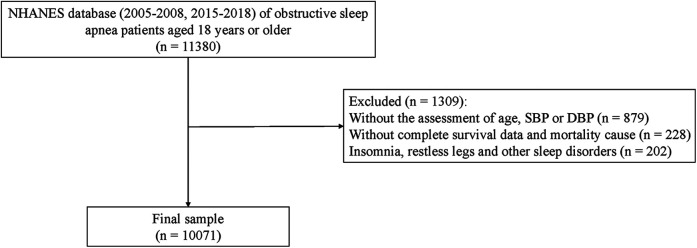
Data filtering flowchart.

This study was a retrospective cohort study, with data sourced from the NHANES database for the years 2005–2008 and 2015–2018, which included specific sleep questionnaire information and blood pressure and survival and death information data. Patient information was collected through interviews and physical examinations during follow-up, which concluded in December 2019, lasting a total of 102.10 ± 2.34 months. Patient records meeting the following criteria were extracted from NHANES: (1) Age ≥ 18 years; (2) Patients with OSA. Records were excluded for patients who possessed the following status: (1) Missing data for age, systolic or diastolic blood pressure; (2) Lacking survival data; (3) With sleep disorders such as insomnia, restless legs syndrome, and other sleep disorders. Specific study population choices are displayed in Figure 1.

### Definition of OSA

The definition of OSA was based on the following three conditions, and the presence of one or more of these conditions was considered diagnostic of OSA (6, 34): (1) Snoring on 3 or more nights per week: Select option 2 or 3 for sleep disorders (SLQ) 030; (2) Snoring, gasping, or experiencing episodes of breath cessation on at least 3 nights per week: Select option 2 or 3 for SLQ040; (3) Feeling extremely sleepy during the day on 16–30 days per month: Select option 4 for SLQ120.

### Evaluation of ePWV

ePWV was calculated based on Equation (1), which was derived from the reference values of the Arterial Stiffness Collaboration (35).

(1) $$\begin{array}{c} \mathrm{ePWV}=9.587-(0.402\times \mathrm{age})+[4.560\times 0.001\times (\mathrm{ag}e^{2})]\\ -[2.621\times 0.00001\times (\mathrm{ag}e^{2})\times \mathrm{MBP}]\\ +(3.176\times 0.001\times \mathrm{age}\times \mathrm{MBP})\\ -(1.832\times 0.01\times \mathrm{MBP}) \end{array}$$

Within this formula, MBP was computed according to Equation (2), where blood pressure was measured and recorded following the protocol published by the American Heart Association (36). Participants were required to remain seated for at least 5 min before testing, and the measurements were taken by trained medical staff. The blood pressure value was the average of three consecutive readings.

(2) MBP=DBP+[0.4×(SBP−DBP)]

### Study outcomes

The outcomes of this study were ACM and CVM. The mortality was determined based on the International Statistical Classification of Diseases and Related Health Problems, 10th Revision (ICD-10) (37). ACM referred to the sum of deaths from all causes. CVM was defined as deaths caused by a range of conditions related to the heart and vascular system. Specifically, in ICD-10, CVM codes included a range of codes related to conditions such as cardiomyopathy (I40–I42), myocarditis (I51.4–I51.6), heart failure (I50), cerebrovascular diseases (I60–I69), and adverse reactions from poisoning or effects on the cardiovascular system (T46).

### Definition of potential variables

According to clinical practice guidelines and the relevant published literature, the following covariates were selected. The latent covariates for the ACM outcome were as follows: Age (years), race, education, marriage, poverty-to-income ratio, smoking (times/week), physical activity (met*minutes/week), total energy (kilocalorie), sleep duration (hours), cardiovascular disease (CVD), diabetes, dyslipidemia, chronic obstructive pulmonary disease (COPD), depression, cancer, aspartate aminotransferase/alanine aminotransferase (AST/ALT) ratio, anti-hypertensive agents, using barbiturates and benzodiazepines, trying to lose weight. The potential covariates for CVM were as follows: Age (years), race, education, marriage, smoking (times/week), physical activity (met*minutes/week), CVD, diabetes, dyslipidemia, COPD, cancer, AST/ALT ratio, uric acid (mg/dl), anti-hypertensive agents, trying to lose weight. According to the subsequent analysis, the final covariate most suitable for inclusion in each outcome was selected. In addition, a specific definition or criterion of each covariate is provided in the supporting material (Supplementary Table S1).

### Statistical analysis

In accordance with the actual conditions of the original database, the existing presence of missing data values was observed, with specific missing variables and proportions detailed in (Supplementary Table S2). In this article, variables with a higher degree of missingness were categorized as unknown. For variables with fewer missing values, a multiple imputation method based on chained equations using a random forest approach was employed to handle the imputation of missing values. After imputation, in order to ensure the reliability of the imputation data, a sensitivity analysis was used to explore whether there was a difference between the missing variable data before and after imputation. The results of the analysis are shown in (Supplementary Table S3), which exhibited that there was no statistical difference between the data before and after imputation.

The study characterized continuous data with mean [SE] and categorical data with case numbers (percentage). Independent *t*-tests compared continuous data, chi-square tests for categorical, and rank sum tests for ordinal data between groups. A weighted univariate Cox regression identified ACM and CVM factors, detailed in (Supplementary Tables S4, S5). Significant factors were used in a weighted Cox regression to assess ePWV's association with ACM and CVM, both as a continuous and quartile variable. The Log-rank test and RCS analyzed survival differences and nonlinear relationships between ePWV and mortality risk. Stratified analyses were based on comorbidities: hypertension, diabetes, dyslipidemia, CVD, and depression.

All statistical tests were conducted using two-sided tests with a significance level of α = 0.05. Data cleaning and handling of missing values were performed using Python 3.9 (https://www.python.org/), while model statistical analysis was carried out using SAS 9.4 (SAS Institute Inc., Cary, NC, USA).

## Results

### Characteristics of the study population

**Figure 2:**
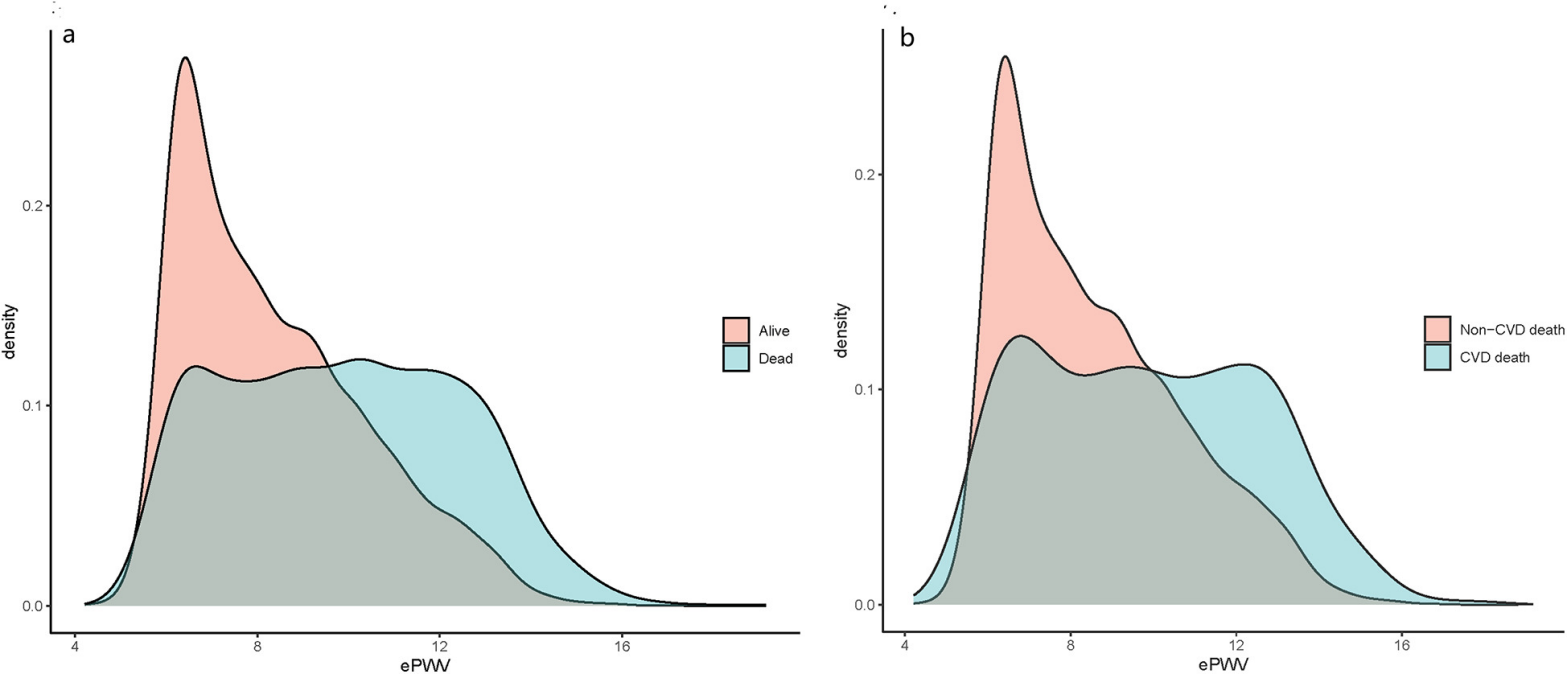
ePWV density distribution curves of OSA patients in different characteristic groups: **(a)** alive and dead population; **(b)** CVD deaths and non-CVD deaths.

This study included a total of 10,071 OSA patients surveyed by NHANES. Among them, 8,440 patients were alive and 1,631 were dead, with 513 experiencing CVD-specific death. The average age of all study population was 48.46 years old, with a male-female ratio close to 1:1. The weighted mean ePWV was 8.31 m/s. The ePWV was categorized into four quartiles as follows: the first quartile ≤ 6.68 m/s (2,457 individuals, 25%), the second quartile 6.68–7.85 m/s (2,127 individuals, 24.99%), the third quartile 7.85–9.49 m/s (2,313 individuals, 25.01%), and the fourth quartile > 9.49 m/s (3,174 individuals, 25.00%). The total population was stratified by survival status, with 8,440 individuals in the survival group and 1,631 in the dead group. Among the dead, there were 513 individuals attributed to CVD-specific death and 9,558 attributed to non-CVD-specific death. The baseline characteristics comparison according to survival status and CVD-specific death are detailed in Supplementary Table S6. The distribution of ePWV among OSA patients is shown in (Supplementary Figure S1), and the stratification of ePWV based on survival status and CVM is depicted in Figures 2a,b. The majority of dead OSA patients and those with CVD-specific death possessed higher ePWV values.

### Associations of ePWV with ACM and CVM

Table 1 and Table 2 showed the results of analyzing the relationship for ePWV with ACM and CVM, respectively. When ePWV was analyzed as a continuous variable, an increase of one unit in ePWV was associated with a 25% increase in the risk of ACM (HR: 1.25, 95% CI: 1.20–1.31) and a 28% increase in the risk of CVD-specific death (HR: 1.28, 95% CI: 1.21–1.36). Additionally, when ePWV was analyzed as a categorical variable, the risk of ACM significantly increased for individuals in the second, third, and fourth quartiles compared to those in the first quartile (ePWV ≤ 6.68 m/s) (all *P* < 0.05). The risks in the aforementioned three groups increased by 32% (HR: 1.32, 95% CI: 2.39–3.98), 74% (HR: 1.74, 95% CI: 1.26–2.40), and 208% (HR: 3.08, 95% CI: 2.39–3.98) compared to the reference group. In association with CVM, the risk of CVD-specific death increased by 76% (HR: 1.76, 95% CI: 1.14–2.73) and 252% (HR: 3.52, 95% CI: 2.20–5.63) for patients in the third and fourth quartiles, respectively, compared to those in the lowest quartile (all *P* < 0.05).

**Table 1:** Association between ePWV and ACM.

Variables	Model 1		Model 2		Model 3	
	HR (95%CI)	*P*	HR (95%CI)	*P*	HR (95%CI)	*P*
ePWV (m/s)	1.34 (1.31–1.38)	<0.001	1.31 (1.26–1.37)	<0.001	1.25 (1.20–1.31)	<0.001
ePWV (m/s)						
≤6.68	Ref		Ref		Ref	
6.68–7.85	1.22 (0.99–1.50)	0.063	1.24 (0.98–1.56)	0.068	1.32 (1.04–1.69)	0.025
7.85–9.49	1.76 (1.36–2.27)	<0.001	1.73 (1.28–2.33)	<0.001	1.74 (1.26–2.40)	0.001
>9.49	4.29 (3.56–5.16)	<0.001	3.68 (2.90–4.66)	<0.001	3.08 (2.39–3.98)	<0.001

Abbreviation: ePWV, estimated pulse wave velocity; CI, confidence interval; HR, hazard ratio. Ref, reference.

Note: Model 1: Adjusted none.

Model 1: Adjusted age, race, education, marriage, poverty-to-income ratio.

Model 2: Adjusted age, race, education, marriage, poverty-to-income ratio, smoke, physical activity, total energy, sleep duration, CVD, diabetes, dyslipidemia, COPD, depression, cancer, AST/ALT ratio, anti-hypertensive agents, barbiturates and benzodiazepines, try to lose weight.

**Table 2:** Association between ePWV and CVM.

Variables	Model 1*		Model 2*		Model 3*	
	HR (95% CI)	*P*	HR (95% CI)	*P*	HR (95% CI)	*P*
ePWV (m/s)	1.34 (1.28–1.39)	<0.001	1.36 (1.29–1.44)	<0.001	1.28 (1.21–1.36)	<0.001
ePWV (m/s)						
≤6.68	Ref		Ref		Ref	
6.68–7.85	1.33 (0.95–1.87)	0.100	1.43 (0.98–2.08)	0.065	1.40 (0.95–2.07)	0.087
7.85–9.49	1.68 (1.10–2.58)	0.018	1.94 (1.30–2.90)	0.002	1.76 (1.14–2.73)	0.012
>9.49	4.27 (2.95–6.17)	<0.001	4.79 (3.01–7.62)	<0.001	3.52 (2.20–5.63)	<0.001

Abbreviation: ePWV, estimated pulse wave velocity; CI, confidence interval; HR, hazard ratio. Ref, reference.

Note: Model 1*: Adjusted none.

Model 1*: Adjusted age, race, education, marriage.

**Figure 3:**
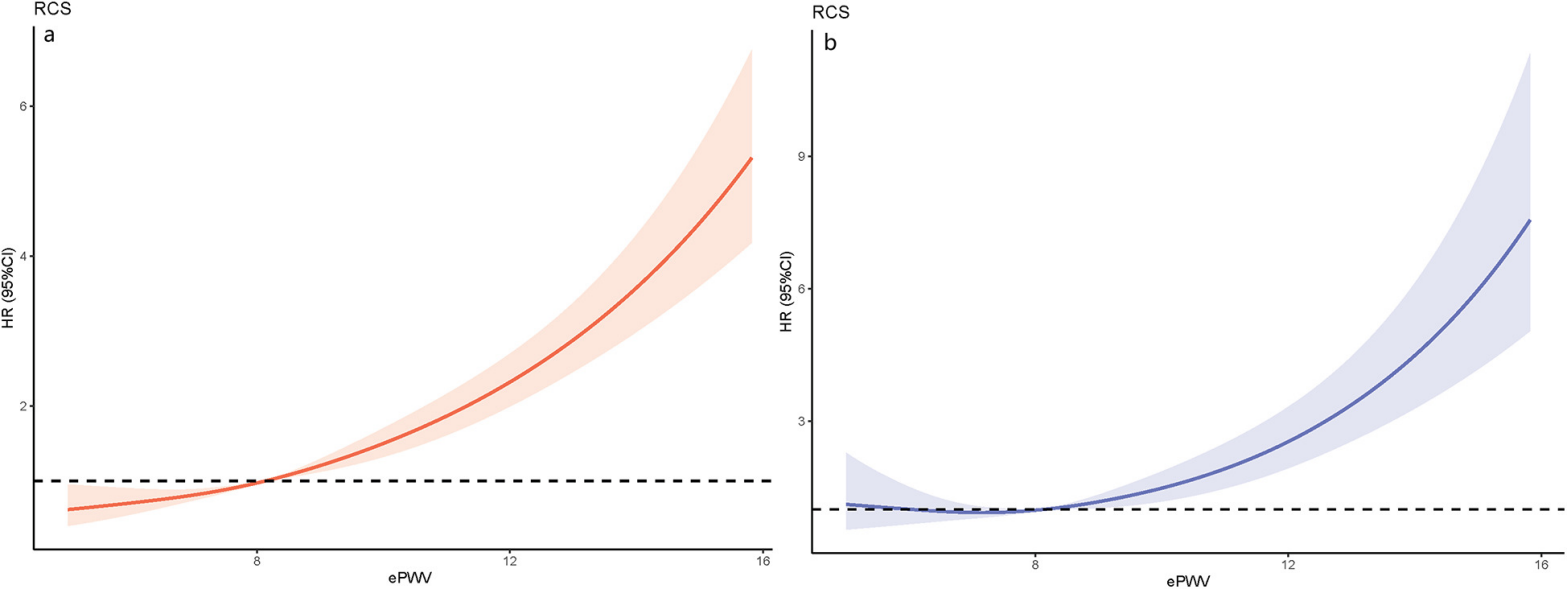
The results of RCS analysis: **(a)** association between ePWV and ACM using a RCS regression; **(b)** association between ePWV and CVM using a RCS regression.

**Figure 4:**
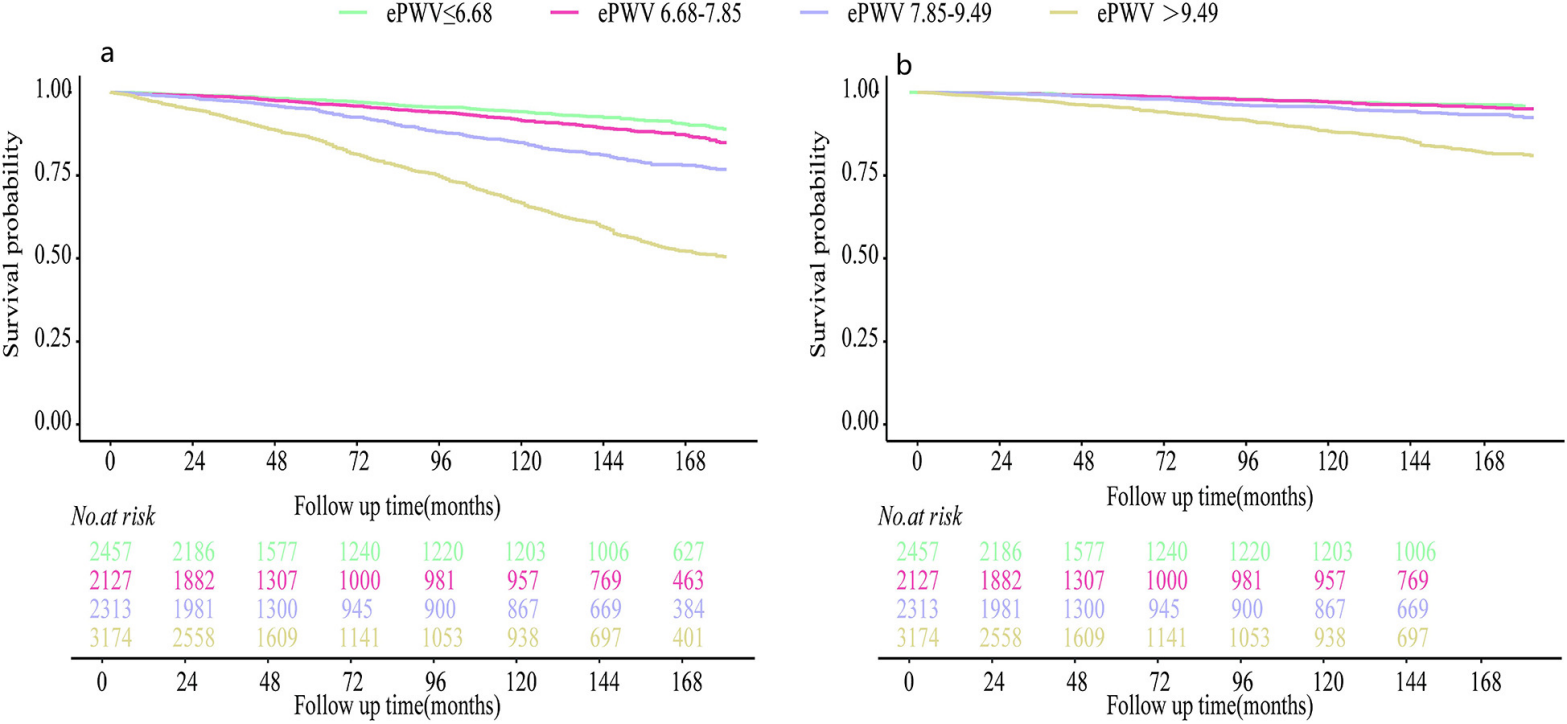
Kaplan–meier survival curves for patients with different degrees of ePWV: **(a)** the outcome variable was all-cause death; **(b)** the outcome variable was CVD-specific death.

Model 2*: Adjusted age, race, education, marriage, smoke, physical activity, CVD, diabetes, dyslipidemia, COPD, cancer, AST/ALT ratio, uric acid, anti-hypertensive agents, try to lose weight. Figures 3a,b visualizes the relationship between the continuous ePWV variable and the risks of ACM and CVM in OSA patients. The results from the RCS model showed that the risks of ACM and CVD-specific death were relatively stable when ePWV was ≤8.1 m/s, and they raised sharply when ePWV exceeded 8.1 m/s. Moreover, Kaplan–Meier (KM) curves also indicated that OSA patients with higher ePWV have significantly increased risks of ACM (Figure 4A) and CVM (Figure 4B). Specifically, when examining the survival profiles between groups of alive and dead, significant differences were observed; better survival was noted for those with ePWV ≤ 6.68 m/s, while the worst survival was observed for those with ePWV > 9.49 m/s. When assessing the survival differences between groups with CVD-specific death and non-CVD-specific death, except for the groups with ePWV ≤ 6.68 m/s and 6.68–7.85 m/s, which showed no significant difference, the other four groups exhibited significant survival disparities. Among them, better survival was observed for those with ePWV ≤ 7.85 m/s, and the worst survival was noted for those with ePWV > 9.49 m/s.

### Stratification analysis of ePWV association with ACM and CVM

As shown in Table 3, the associations of ePWV with ACM and CVM were explored in subgroups defined by the presence of diabetes, hypertension, dyslipidemia, CVD, and depression. When ePWV was treated as a continuous variable, a statistically significant association between ePWV and both ACM and CVM was observed across all subgroups, indicating that ePWV was positively correlated with ACM and CVM. The analysis results of ePWV as a categorical variable in relation to ACM and CVM are specifically presented in Table 3.

**Table 3:** Subgroup analysis for association of ePWV with ACM/CVM.

ACM					CVM				
Subgroup	HR (95% CI)	*P*	HR (95% CI)	*P*		HR (95% CI)	*P*	HR (95% CI)	*P*
**Subgroup I: Diabetes**	None (*n* = 7,836)		Yes (*n* = 2,235)		Subgroup I*: Diabetes	No (*n* = 7,836)		Yes (*n* = 2,235)	
ePWV (m/s)	1.27 (1.20–1.34)	<0.001	1.23 (1.15–1.32)	<0.001	ePWV (m/s)	1.34 (1.24–1.46)	<0.001	1.14 (1.05–1.24)	0.002
≤6.68	Ref		Ref		≤6.68	Ref		Ref	
6.68–7.85	1.30 (1.01–1.66)	0.042	1.59 (0.68–3.73)	0.282	6.68–7.85	1.45 (0.97–2.17)	0.072	1.61 (0.50–5.18)	0.416
7.85–9.49	1.81 (1.28–2.57)	0.001	1.72 (0.71–4.18)	0.224	7.85–9.49	1.70 (1.11–2.59)	0.015	2.47 (0.84–7.28)	0.098
>9.49	3.28 (2.38–4.53)	<0.001	2.97 (1.18–7.47)	0.022	>9.49	3.99 (2.35–6.78)	<0.001	3.24 (0.92–11.37)	0.066
**Subgroup II: Hypertension**	No (*n* = 4,961)		Yes (*n* = 5,110)		Subgroup II*: Hypertension	No (*n* = 4,961)		Yes (*n* = 5,110)	
ePWV (m/s)					ePWV (m/s)				
≤6.68	Ref		Ref		≤6.68	Ref		Ref	
6.68–7.85	1.23 (0.93–1.62)	0.144	1.36 (0.75–2.47)	0.310	6.68–7.85	1.53 (1.02–2.29)	0.041	1.62 (0.74–3.54)	0.220
7.85–9.49	1.78 (1.11–2.85)	0.018	1.53 (0.84–2.78)	0.163	7.85–9.49	1.99 (0.95–4.18)	0.069	1.99 (0.78–5.09)	0.146
>9.49	4.34 (2.61–7.21)	<0.001	2.45 (1.41–4.27)	0.002	>9.49	4.10 (1.84–9.14)	<0.001	3.77 (1.32–10.74)	0.014
**Subgroup III: Dyslipidemia**	No (*n* = 2,770)		Yes (*n* = 7,301)		Subgroup III*: Dyslipidemia	No (*n* = 2,770)		Yes (*n* = 7,301)	
ePWV (m/s)	1.31 (1.18–1.44)	<0.001	1.24 (1.19–1.30)	<0.001	ePWV (m/s)	1.21 (1.01–1.45)	0.040	1.30 (1.22–1.38)	<0.001
≤6.68	Ref		Ref		≤6.68	Ref		Ref	
6.68–7.85	1.41 (0.89–2.21)	0.138	1.29 (0.91–1.83)	0.156	6.68–7.85	1.56 (0.77–3.16)	0.215	1.34 (0.81–2.22)	0.244
7.85–9.49	1.84 (0.96–3.52)	0.066	1.70 (1.17–2.47)	0.006	7.85–9.49	1.82 (0.76–4.36)	0.175	1.71 (1.07–2.76)	0.027
>9.49	3.82 (1.99–7.32)	<0.001	2.94 (2.19–3.94)	<0.001	>9.49	2.42 (0.92–6.34)	0.072	3.63 (2.15–6.11)	<0.001
**Subgroup IV: CVD**	No (*n* = 7,687)		Yes (*n* = 2,384)		Subgroup IV*: CVD	No (*n* = 7,687)		Yes (*n* = 2,384)	
ePWV (m/s)	1.32 (1.25–1.39)	<0.001	1.19 (1.11–1.27)	<0.001	ePWV (m/s)	1.36 (1.22–1.51)	<0.001	1.21 (1.08–1.34)	0.001
≤6.68	Ref		Ref		≤6.68	Ref		Ref	
6.68–7.85	1.32 (1.03–1.69)	0.028	0.79 (0.34–1.81)	0.569	6.68–7.85	1.45 (0.99–2.14)	0.059	1.02 (0.20–5.20)	0.983
7.85–9.49	1.78 (1.27–2.49)	0.001	1.08 (0.46–2.56)	0.856	7.85–9.49	1.81 (1.21–2.69)	0.004	1.40 (0.20–9.89)	0.734
>9.49	3.63 (2.57–5.13)	<0.001	1.60 (0.70–3.66)	0.257	>9.49	4.00 (2.25–7.11)	<0.001	2.42 (0.27–21.42)	0.422
**Subgroup V: Depression**	No (*n* = 8,108)		Yes (*n* = 1,963)		Subgroup V*: Depression	No (*n* = 8,108)		Yes (*n* = 1,963)	
ePWV (m/s)	1.23 (1.17–1.29)	<0.001	1.36 (1.27–1.46)	<0.001	ePWV (m/s)	1.25 (1.17–1.34)	<0.001	1.41 (1.17–1.69)	<0.001
≤6.68	Ref		Ref		≤6.68	Ref		Ref	
6.68–7.85	1.44 (1.09–1.90)	0.011	0.98 (0.55–1.74)	0.943	6.68–7.85	1.51 (1.01–2.26)	0.046	1.04 (0.36–2.99)	0.948
7.85–9.49	1.93 (1.39–2.67)	<0.001	1.28 (0.60–2.72)	0.519	7.85–9.49	1.99 (1.30–3.04)	0.002	1.04 (0.29–3.78)	0.953
>9.49	3.16 (2.27–4.40)	<0.001	2.77 (1.37–5.60)	0.005	>9.49	3.58 (2.21–5.81)	<0.001	2.85 (0.56–14.42)	0.202

Abbreviation: ACM, all-cause mortality; CI, confidence interval; CVM, cardiovascular disease mortality; ePWV, estimated pulse wave velocity; HR, hazard ratio; Ref, reference. **Subgroup I** adjusted: Age, race, education, marriage, poverty-to-income ratio, smoke, physical activity, total energy, sleep duration, CVD, dyslipidemia, COPD, depression, cancer, AST/ALT ratio, anti-hypertensive agents, barbiturates and benzodiazepines, try to lose weight. **Subgroup II** adjusted: Age, race, education, marriage, poverty-to-income ratio, smoke, physical activity, total energy, sleep duration, CVD, diabetes, dyslipidemia, COPD, depression, cancer, AST/ALT ratio, anti-hypertensive agents, barbiturates and benzodiazepines, try to lose weight. **Subgroup III** adjusted: Age, race, education, marriage, poverty-to-income ratio, smoke, physical activity, total energy, sleep duration, CVD, diabetes, COPD, depression, cancer, AST/ALT ratio, anti-hypertensive agents, barbiturates and benzodiazepines, try to lose weight. **Subgroup IV** adjusted: Age, race, education, marriage, poverty-to-income ratio, smoke, physical activity, total energy, sleep duration, diabetes, dyslipidemia, COPD, depression, cancer, AST/ALT ratio, anti-hypertensive agents, barbiturates and benzodiazepines, try to lose weight. **Subgroup V** adjusted: Age, race, education, marriage, poverty-to-income ratio, smoke, physical activity, total energy, sleep duration, CVD, diabetes, dyslipidemia, COPD, cancer, AST/ALT ratio, anti-hypertensive agents, barbiturates and benzodiazepines, try to lose weight.

Subgroup I* adjusted: Age, race, education, marriage, smoke, physical activity, CVD, dyslipidemia, COPD, cancer, AST/ALT ratio, uric acid, anti-hypertensive agents, try to lose weight. Subgroup II* adjusted: Age, race, education, marriage, smoke, physical activity, CVD, diabetes, dyslipidemia, COPD, cancer, AST/ALT ratio, uric acid, anti-hypertensive agents, try to lose weight. Subgroup III* adjusted: Age, race, education, marriage, smoke, physical activity, CVD, diabetes, COPD, cancer, AST/ALT ratio, uric acid, anti-hypertensive agents, try to lose weight. Subgroup IV* adjusted: Age, race, education, marriage, smoke, physical activity, diabetes, dyslipidemia, COPD, cancer, AST/ALT ratio, uric acid, anti-hypertensive agents, try to lose weight. Subgroup V* adjusted: Age, race, education, marriage, smoke, physical activity, CVD, diabetes, dyslipidemia, COPD, cancer, AST/ALT ratio, uric acid, anti-hypertensive agents, try to lose weight.

## Discussion

OSA is significantly associated with CVD occurrence and mortality (38). Arterial stiffness (AS), a known cardiovascular risk factor, is linked to OSA-related cardiovascular complications (39). Studies show a correlation between AS severity and OSA, suggesting OSA's role in AS development and associated CVD risks (40, 41). This study used ePWV, an indicator of AS, to explore its link with ACM and CVD-specific death in OSA patients. The results revealed a positive correlation between ePWV and both ACM and CVM, indicating that higher ePWV levels increase the risk of all-cause or CVD-related death in OSA patients (15).

Heffernan et al. (31) found ePWV linked to cardiovascular (CVD) and all-cause mortality (ACM) in a broad U.S. adult sample, suggesting it could provide insights into CVD and ACM risk beyond traditional risk factors. Huang et al. (12) and Wu et al. (17) found higher ePWV associated with increased ACM and cardio-cerebrovascular disease (CCD) mortality in stroke patients and diabetic patients, respectively, indicating ePWV as an independent risk factor. Similar results were seen in studies on hypertension and coronary artery disease patients (32, 42). In OSA patients, higher ePWV levels also correlated with increased ACM and CVM risks, regardless of confounding factors. Li's study (43) showed ePWV's strong predictive value for CVM and ACM in obese individuals, proposing it as a potential biomarker for mortality risk assessment. ePWV, derived from age and blood pressure, could be a valuable CVD risk indicator, especially in OSA patients, and its inclusion in routine health checks could aid in understanding and managing CVD risk across populations.

In patients with OSA, the intima-media thickness and carotid artery diameter are generally higher, and numerous studies have confirmed the association between OSA and vascular dysfunction (44, 45). The aforementioned linkage can be attributed to the intermittent hypoxia often experienced by OSA patients, leading to the occurrence of intermittent hypoxemia (46). Intermittent hypoxia tends to induce an increase in inflammatory substances such as high-sensitivity C-reactive protein and interleukin-6, causing an inflammatory response (47). These inflammatory substances are closely related to endothelial function and promote the occurrence of arteriosclerosis, leading to the development of CVD (30). Intermittent hypoxia also significantly increases the production of reactive oxygen species, leading to increased oxidative stress (48). Oxidative stress reduces the generation of nitric oxide, causing endothelial dysfunction and arteriosclerosis, ultimately increasing CVD in OSA patients (49, 50). This study used ePWV, a simple assessment indicator of arteriosclerosis, to observe the association between arteriosclerosis and ACM and CVM in OSA patients, finding a close positive correlation of ePWV with ACM and CVM. Therefore, early prevention of arteriosclerosis is very important for the OSA population. In summary, ePWV can serve as a widespread screening and self-monitoring indicator for the OSA population and even the general population. Early attention to arteriosclerosis may help the OSA population better prevent CVD, providing some assurance for the extension of healthy life expectancy in the OSA population.

Stratified analysis showed that in OSA patients with comorbidities like diabetes, dyslipidemia, hypertension, CVD, or depression, higher ePWV was linked to increased all-cause and CVD-specific mortality risks (51–54). This trend was also seen in OSA patients without these conditions, though the mechanisms are not fully understood. ePWV is a common indicator for assessing arterial stiffness (AS) (43), which can predict cardiovascular damage or prognosis by estimating the load on coronary, cerebral arteries, and aorta (55, 56). Epidemiological evidence supports arteriosclerosis's role in diseases like diabetes, dyslipidemia, hypertension, CVD, and depression (51–54). Possible reasons for subgroup analysis findings include: increased pulse pressure from arterial stiffness leading to vascular damage and mortality (12, 57); OSA patients with these diseases being more frail, obscuring the ePWV-mortality link; and early intervention reducing death risk, potentially diminishing ePWV's association with ACM and CVM (43). For instance, SGLT2 inhibitors like dapagliflozin can reduce PWV in diabetic patients (58), and their use is associated with lower cardiovascular event risks (59).

This study has certain strengths. Firstly, to our knowledge, this was the first study to explore the relationship between ePWV and ACM/CVM in patients with OSA, providing new perspectives for the screening and management of CVD prevention in this high-risk population. Secondly, the study population was drawn from the NHANES database, which employed a multi-stage complex sampling method, ensuring a good representation of the local population. Lastly, the large sample size and sufficient follow-up time of this survey ensured the occurrence of outcome events, providing robust statistical power. Despite some significant findings, the study possessed certain limitations. First, due to the limitations of the database, the study population was identified based on typical symptoms of OSA collected through questionnaires, lacking data related to polysomnography or sleep apnea tests, which may lead to selection bias and information bias. Therefore, future studies should use more objective measurement data to verify the aforementioned associations. Second, given that the database lacks follow-up data pertaining to ventilator treatment, this study did not delve into this aspect. This may impact the prognosis of OSA patients to a certain extent and thus should be taken into consideration when interpreting the conclusions. Third, although this study was a cohort study, it was also subject to inherent biases associated with this type of research, and future explorations could be conducted in studies with higher methodological rigor, such as randomized controlled trials. Fourth, Our study extends the application of the estimated pulse wave velocity (ePWV) equation to populations with cardiovascular disease (CVD), diabetes, and antihypertensive therapy—groups explicitly excluded from the original derivation cohort. While the generalizability of the ePWV formula in such populations may be questioned, our findings suggest that its predictive validity remains robust. This can be attributed to the pathophysiological primacy of age and blood pressure (BP) in driving arterial stiffness, which collectively explain over 80% of the variance in directly measured carotid-femoral PWV, even in high-risk populations. Importantly, the use of measured BP values (rather than hypothetical “untreated” BP) in ePWV calculation inherently accounts for the effects of antihypertensive therapy, reflecting the net arterial health status under real-world clinical management. Nevertheless, the original equation does not explicitly incorporate diabetes-specific pathways (e.g., advanced glycation end-product accumulation) or CVD-related vascular remodeling, which may partially attenuate its precision in these subgroups. Future studies integrating comorbidity-adjusted ePWV models with direct PWV measurements could further refine risk stratification in complex populations.Lastly, the study data came from the NHANES database of American adults, so caution should be exercised when generalizing the results to populations of different races or with different economic and geographical conditions, and further research is needed to interpret these results in other populations.

## Conclusion

This study found that among 10,071 OSA patients surveyed by NHANES from 2005 to 2008 and 2015 to 2018, higher levels of ePWV during the follow-up period were associated with increased ACM and CVM. This research provides new scientific evidence for the future clinical use of ePWV as an initial assessment of AS and as a screening tool for the early identification of high-risk populations.

## Data Availability

The datasets presented in this study can be found in online repositories. The names of the repository/repositories and accession number(s) can be found in the article/Supplementary Material.
