# Supplementary material for: Association of estimated pulse wave velocity with all-cause mortality and cardiovascular mortality in obstructive sleep apnea patients: results from NHANES
Source: Front Cardiovasc Med. 2025 Jun 12;12:1571610. doi: 10.3389/fcvm.2025.1571610 (PMC12198205; doi:10.3389/fcvm.2025.1571610)
Supplement: Supplementary file 1 [file Datasheet1.pdf]

Table S1 Definitions or criterions of covariates

Covariates	Definitions/scoring rule
Physical activity	Physical activity can be converted to energy expenditure, which was calculated based on the "PAQ" physical activity report in the database questionnaire, energy expenditure (MET-min) = recommended metabolic equivalent (MET) × exercise time (min) of the corresponding activity, which can be converted to weekly energy expenditure. Energy expenditure (MET-min) = recommended metabolic equivalent (MET) × exercise time (min) of the corresponding activity, which can be converted to weekly energy expenditure, was grouped according to whether it was < 450 MET-min/week or not, and was categorized as < 450 MET-min/week, ≥ 450 MET-min/week, and Unknown.
Total energy	Diets + Supplements.
Sleep duration	Sleep length: SLD010H (2005-2008) or SLD012 (2015-2018), divided by ≤ 6 h, 6-9 h, and ≥ 9 h.
CVD	Cardiovascular disease (self-reported coronary heart disease, angina, heart failure, heart attack, stroke, or cardiovascular drug code): "Ever told you had angina or heart failure" (MCQ160D) or "Ever told you had heart attack" (MCQ160E) or "Has a doctor or other health professional ever told you that you had coronary heart disease "(MCQ160C) or "Ever told you had a stroke" (MCQ160F) or "Ever told had congestive heart failure "(MCQ160B); or cardiovascular disease drug code First Level Category ID-Name for or cardiovascular disease drug code 40-CARDIOVASCULAR AGENTS-41, 43, 44, 45, 46, 50, 51, 52, 53, 54, 56, 303 340, 342, 430, 433, 483.
Diabetes	HbA1c ≥ 6.5% or fasting glucose ≥ 126 mg/dL or 2 h OGTT glucose ≥ 200 or self-reported diagnosis of diabetes DIQ010 - Doctor told you have diabetes or use of insulin (DIQ050) or hypoglycemic agents (DIQ070 or hypoglycemic agent code 358- metabolic agents-99-antidiabetic agent).
Dyslipidemia	TC ≥ 200 mg/dL (5.2 mmol/L) or TG ≥ 150 mg/dL (1.7 mmol/L) or LDL-C ≥ 130 mg/dL (3.4 mmol/L) or HDL-C ≤ 40 mg/dL (1.0 mmol/L) [PMID: 22522068] or self-reported hypercholesterolemia (BPQ080) or receiving cholesterol-lowering therapy (BPQ090D) or lipid-lowering drugs (358-metabolic agents-19-antihyperlipemic agents).
COPD	MCQ160o - Ever told you had COPD?
Depression	PHQ-9 scale, ≥ 10 for depression or taking antidepressants: class I ID 242 under class II ID 249 in RXQ_DRUG.
Cancer	MCQ220 - Ever told you had cancer or malignancy
AST/ALT ratio	LBXSASSI Aspartate aminotransferase AST (U/L), LBXSATSI-Alanine aminotransferase ALT (U/L), the ratio of the two indicators.
Anti-hypertensive agents	Antihypertensive drug BPQ040A option 1 or BPQ050A option 1 or with the following codes 40-CARDIOVASCULAR AGENTS-42, 47, 48, 49, 482, 55.
Barbiturates and benzodiazepines	Barbiturates and benzodiazepines: drug codes I 57 - II 67 - III 68, 69.
Try to lose weight	WHQ070 - Tried to lose weight in past year.

BMI	Classified according to < 25, 25-30, ≥ 30 kg/m^2 based on guidelines issued by WHO.
Hypertension	Hypertension was determined by SBP ≥140 mmHg and/or DBP ≥90 mmHg; or being diagnosed with hypertension BPQ020 choice 1 or BPQ030 choice 1 or taking antihypertensive medication BPQ040A choice 1 or BPQ050A choice 1 or having the following codes 40-CARDIOVASCULAR AGENTS-42, 47, 48 , 49, 482, 55 (given covariance with exposure, hypertension was used only as a basis for subgroup delineation and not as a covariate)

Abbreviation: ALT: Alanine aminotransferase; AST: Aspartate aminotransferase; BMI: Body mass index; BPQ: Blood pressure questionnaire; COPD: Chronic obstructive pulmonary disease; CVD: Cardiovascular disease; DBP: Diastolic blood pressure; DIQ: Diabetes interview questionnaire; HbA1c: Hemoglobin A1c; HDL-C: High-density lipoprotein cholesterol; LDL-C: Low-density lipoprotein cholesterol; MCQ: Medical conditions questionnaire; OGTT: Oral glucose tolerance test; PAQ: Physical activity grouping; PHQ: Patient health questionnaire; RXQ_DRUG: Prescription medications questionnaire; SBP: Systolic blood pressure; SLD: Sleep duration; TC: Total cholesterol; TG: Triglycerides; WHO: World health organization; WHQ: Weight History questionnaire.

Table S2 The details of the missing variable

Missing variable name	Missing data count and proportion
Education	376 (3.73%)
Marriage	240 (2.38%)
Poverty-to-income ratio	890 (8.84%)
Smoke	224 (2.22%)
Drink	1013 (10.06%)
Physical activity	3080 (30.58%)
Total energy	538 (5.34%)
Sleep duration	42 (0.42%)
COPD	4950 (49.15%)
Depression	639 (6.34%)
BMI	135 (1.34%)
WBC	452 (4.49%)
AST	585 (5.81%)
ALT	574 (5.7%)
Creatinine	547 (5.43%)
Uric acid	551 (5.47%)
Try to lose weight	1361 (13.51%)

Abbreviation: ALT: Alanine aminotransferase; AST: Aspartate aminotransferase; BMI: Body mass index; COPD: Chronic obstructive pulmonary disease; WBC: White blood cell.

Table S3 Sensitivity analysis

Variables	Before interpolation	After interpolation	Statistics	*P*
Education, n (%)			$\chi^2 = 0.29$	0.589
High school and below	4891 (42.10)	5067 (42.13)		
University and above	4804 (57.90)	5004 (57.87)		
Marriage, n (%)			$\chi^2 = 2.08$	0.353
Married	5586 (60.42)	5721 (60.34)		
Never married	1412 (13.68)	1443 (13.70)		
Others	2833 (25.90)	2907 (25.96)		
Pir, n (%)			$\chi^2 = 4.45$	0.108
≤ 1.3	2681 (19.33)	2971 (19.59)		
1.3-3.5	3700 (37.04)	4079 (37.08)		
> 3.5	2800 (43.62)	3021 (43.33)		
Smoke, n (%)			$\chi^2 = 1.25$	0.264
No	5121 (50.65)	5275 (50.70)		
Yes	4726 (49.35)	4796 (49.30)		
Drink, n (%)			$\chi^2 = 0.70$	0.403
≤ 1time/week	7256 (75.25)	8116 (75.41)		
> 1 time/week	1802 (24.75)	1955 (24.59)		
Total energy, kcal, Mean (S.E)	2268.80 (15.28)	2265.09 (14.50)	t = 1.71	0.092
Sleep duration, hours, Mean (S.E)	7.18 (0.02)	7.19 (0.02)	t = -0.51	0.615
Depression, n (%)			$\chi^2 = 0.00$	0.990
No	7609 (79.15)	8108 (79.14)		
Yes	1823 (20.85)	1963 (20.86)		
BMI, kg/m^2, Mean (S.E)	30.65 (0.12)	30.65 (0.11)	t = -0.15	0.883
WBC, 1000 cells/uL, Mean (S.E)	7.52 (0.04)	7.53 (0.04)	t = -1.18	0.242
ALT, U/L, Mean (S.E)	26.76 (0.23)	26.81 (0.22)	t = -0.79	0.432
AST, U/L, Mean (S.E)	25.24 (0.19)	25.32 (0.19)	t = -1.39	0.170
Creatinine, mg/dL, Mean (S.E)	0.90 (0.00)	0.90 (0.00)	t = -1.83	0.073
Uric acid, mg/dL, Mean (S.E)	5.60 (0.02)	5.60 (0.02)	t = 0.34	0.735

Abbreviation: ALT: Alanine aminotransferase; AST: Aspartate aminotransferase; BMI: Body mass index; Pir: Poverty-to-income ratio; S.E: Standard error. Note: t indicates t-test; $\chi 2$, chi-square test.

Table S4 Weighted univariate COX regression model for ACM

Variables	HR (95%CI)	*P*
Age, years		
< 45	Ref	
≥ 45	2.28 (1.97-2.64)	**<0.001**
Gender		
Male	Ref	
Female	0.96 (0.85-1.08)	0.493
Race		
Non-Hispanic White	Ref	
Non-Hispanic Black	0.83 (0.70-0.99)	**0.036**
Others	0.67 (0.58-0.77)	**<0.001**
Education		
High school and below	Ref	
University and above	0.70 (0.62-0.80)	**<0.001**
Marriage		
Married	Ref	
Never married	0.64 (0.52-0.78)	**<0.001**
Others	1.16 (1.01-1.35)	**0.043**
Pir		
≤ 1.3	Ref	
1.3-3.5	0.99 (0.84-1.16)	0.878
> 3.5	0.65 (0.53-0.81)	**<0.001**
Smoke		
No	Ref	
Yes	1.41 (1.23-1.62)	**<0.001**
Drink		
≤ 1time/week	Ref	
> 1time/week	0.88 (0.76-1.03)	0.108
Physical activity		
<450 met*minutes/week	Ref	
≥450 met*minutes/week	0.97 (0.80-1.19)	0.803
Unknown	1.54 (1.25-1.90)	**<0.001**
Total energy	0.99 (0.99-0.99)	**0.008**
Sleep duration, hours		
6-9	Ref	
≤ 6	1.02 (0.92-1.14)	0.693
≥ 9	1.44 (1.19-1.74)	**<0.001**
CVD		
No	Ref	
Yes	2.65 (2.27-3.09)	**<0.001**
Diabetes		
No	Ref	
Yes	2.01 (1.77-2.27)	**<0.001**
Dyslipidemia		
No	Ref	

Yes	1.21 (1.04-1.41)	**0.015**
COPD		
No	Ref	
Yes	6.55 (4.07-10.54)	**<0.001**
Unknown	1.84 (1.31-2.59)	**<0.001**
Depression		
No	Ref	
Yes	1.37 (1.16-1.62)	**<0.001**
Cancer		
No	Ref	
Yes	2.08 (1.73-2.51)	**<0.001**
BMI, kg/m^2		
< 25	Ref	
25-30	0.89 (0.74-1.07)	0.203
≥ 30	0.90 (0.76-1.08)	0.255
WBC	1.02 (1.01-1.04)	0.065
AST/ALT ratio	2.17 (1.82-2.58)	**<0.001**
Creatinine	1.00 (0.76-1.32)	1.000
Uric acid	1.06 (1.01-1.11)	0.053
Anti-hypertensive agents		
No	Ref	
Yes	2.19 (1.90-2.53)	**<0.001**
Barbiturates and benzodiazepines		
No	Ref	
Yes	1.63 (1.22-2.17)	**0.001**
Try to lose weight		
No	Ref	
Yes	0.72 (0.63-0.81)	**<0.001**
Unknown	0.71 (0.56-0.92)	**0.010**

Abbreviation: ALT: Alanine aminotransferase; AST: Aspartate aminotransferase; BMI: Body mass index; CI: confidence interval; COPD: Chronic obstructive pulmonary disease; CVD: Cardiovascular disease; HR: Hazards ratio; Pir: Poverty-to-income ratio; Ref: Reference; WBC: White blood cell.

Table S5 Weighted univariate COX regression model for CVM

Variables	HR (95%CI)	*P*
Age, years		
< 45	Ref	
≥ 45	2.00 (1.47-2.71)	<0.001
Gender		
Male	Ref	
Female	0.84 (0.69-1.04)	0.104
Race		
Non-Hispanic White	Ref	
Non-Hispanic Black	0.86 (0.62-1.20)	0.367
Others	0.74 (0.58-0.93)	0.011
Education		
High school and below	Ref	
University and above	0.67 (0.55-0.82)	<0.001
Marriage		
Married	Ref	
Never married	0.63 (0.42-0.95)	0.027
Others	0.97 (0.76-1.23)	0.785
Pir		
≤ 1.3	Ref	
1.3-3.5	1.21 (0.90-1.62)	0.201
> 3.5	0.94 (0.67-1.33)	0.733
Smoke		
No	Ref	
Yes	1.31 (1.05-1.64)	0.019
Drink		
≤ 1 time/week	Ref	
> 1 time/week	0.90 (0.69-1.18)	0.443
Physical activity		
< 450 met*minutes/week	Ref	
≥ 450 met*minutes/week	0.83 (0.59-1.19)	0.305
Unknown	1.44 (1.05-1.97)	0.025
Total energy	0.99 (0.99-0.99)	0.940
Sleep duration, hours		
6-9	Ref	
≤ 6	0.93 (0.73-1.20)	0.589
≥ 9	1.44 (0.97-2.14)	0.072
CVD		
No	Ref	
Yes	2.65 (1.95-3.58)	<0.001
Diabetes		
No	Ref	
Yes	1.94 (1.51-2.49)	<0.001
Dyslipidemia		
No	Ref	

Yes	1.51 (1.18-1.93)	0.001
COPD		
No	Ref	
Yes	6.63 (1.85-23.73)	0.004
Unknown	2.54 (1.34-4.85)	0.005
Depression		
No	Ref	
Yes	1.28 (0.99-1.65)	0.060
Cancer		
No	Ref	
Yes	2.17 (1.58-2.96)	<0.001
BMI kg/m^2		
< 25	Ref	
25 - 30	1.09 (0.78-1.53)	0.590
≥ 30	1.06 (0.75-1.51)	0.734
WBC	1.01 (0.97-1.05)	0.679
AST/ALT ratio	1.76 (1.30-2.39)	<0.001
Creatinine	1.00 (0.73-1.38)	1.000
Uric acid	1.10 (1.02-1.18)	0.020
Anti-hypertensive agents		
No	Ref	
Yes	2.23 (1.72-2.91)	<0.001
Barbiturates and benzodiazepines		
No	Ref	
Yes	1.58 (0.91-2.74)	0.104
Try to lose weight		
No	Ref	
Yes	0.93 (0.73-1.17)	0.509
Unknown	0.70 (0.49-0.99)	0.045

Abbreviation: ALT: Alanine aminotransferase; AST: Aspartate aminotransferase; BMI: Body mass index; CI: confidence interval; COPD: Chronic obstructive pulmonary disease; CVD: Cardiovascular disease; HR: Hazards ratio; Pir: Poverty-to-income ratio; Ref: Reference; WBC: White blood cell.

Table S6 Demographic characteristics and physiological status information

Variables	Total (n = 10071)	Alive (n = 8440)	Dead (n = 1631)	Statistics	*P*	Non-CVD deaths (n = 9558)	CVD deaths (n = 513)	Statistics	*P*
Age, years, Mean (S.E)	48.46 (0.32)	47.19 (0.32)	56.47 (0.60)	t = -16.46	< 0.001	48.11 (0.32)	56.07 (0.98)	t = -8.10	< 0.001
Age, n (%)				χ^2 = 107.00	< 0.001			χ^2 = 15.42	< 0.001
<45	3921 (41.74)	3568 (44.06)	353 (27.11)			3793 (42.30)	128 (29.76)		
≥45	6150 (58.26)	4872 (55.94)	1278 (72.89)			5765 (57.70)	385 (70.24)		
Gender, n (%)				χ^2 = 3.49	0.062			χ^2 = 5.87	0.015
Male	5448 (54.84)	4491 (54.46)	957 (57.25)			5142 (54.59)	306 (60.31)		
Female	4623 (45.16)	3949 (45.54)	674 (42.75)			4416 (45.41)	207 (39.69)		
Race, n (%)				χ^2 = 36.41	< 0.001			χ^2 = 10.71	0.005
Non-Hispanic White	3990 (66.60)	3095 (65.24)	895 (75.19)			3714 (66.26)	276 (73.88)		
Non-Hispanic Black	2200 (11.24)	1865 (11.37)	335 (10.44)			2093 (11.27)	107 (10.57)		
Others	3881 (22.16)	3480 (23.39)	401 (14.37)			3751 (22.46)	130 (15.56)		
Education, n (%)				χ^2 = 37.55	< 0.001			χ^2 = 22.05	< 0.001
High school and below	5067 (42.13)	4056 (40.37)	1011 (53.28)			4752 (41.56)	315 (54.37)		
University and above	5004 (57.87)	4384 (59.63)	620 (46.72)			4806 (58.44)	198 (45.63)		
Marriage, n (%)				χ^2 = 23.78	< 0.001			χ^2 = 7.99	0.018
Married	5721 (60.34)	4758 (60.03)	963 (62.30)			5412 (60.09)	309 (65.56)		
Never married	1443 (13.70)	1293 (14.47)	150 (8.85)			1392 (13.91)	51 (9.23)		
Others	2907 (25.96)	2389 (25.50)	518 (28.85)			2754 (26.00)	153 (25.21)		
Pir, n (%)				χ^2 = 19.60	< 0.001			χ^2 = 3.69	0.158
≤ 1.3	2971 (19.59)	2483 (19.34)	488 (21.18)			2846 (19.72)	125 (16.91)		
1.3-3.5	4079 (37.08)	3334 (36.13)	745 (43.12)			3833 (36.85)	246 (42.05)		
> 3.5	3021 (43.33)	2623 (44.53)	398 (35.70)			2879 (43.43)	142 (41.03)		
Smoke, n (%)				χ^2 = 26.10	< 0.001			χ^2 = 6.86	0.009

No	5275 (50.70)	4582 (52.12)	693 (41.75)			5039 (51.04)	236 (43.53)		
Yes	4796 (49.30)	3858 (47.88)	938 (58.25)			4519 (48.96)	277 (56.47)		
Drink, n (%)				$\chi^2 = 1.41$	0.235			$\chi^2 = 0.31$	0.580
≤ 1time/week	8116 (75.41)	6803 (75.15)	1313 (77.05)			7705 (75.35)	411 (76.67)		
> 1time/week	1955 (24.59)	1637 (24.85)	318 (22.95)			1853 (24.65)	102 (23.33)		
Physical activity, n (%)				$\chi^2 = 94.07$	< 0.001			$\chi^2 = 49.17$	< 0.001
< 450 met*minutes/week	1625 (17.37)	1291 (16.56)	334 (22.48)			1513 (17.04)	112 (24.38)		
≥ 450 met*minutes/week	5366 (57.14)	4754 (59.83)	612 (40.16)			5194 (58.05)	172 (37.55)		
Unknown	3080 (25.49)	2395 (23.61)	685 (37.35)			2851 (24.91)	229 (38.06)		
Total energy, kcal, Mean (S.E)	2265.09 (14.50)	2278.18 (14.98)	2182.47 (42.55)	t = 2.16	0.035	2263.53 (14.36)	2298.46 (85.49)	t = -0.41	0.685
Sleep duration, n (%)				$\chi^2 = 68.56$	< 0.001			$\chi^2 = 9.95$	0.007
6-9 h	5490 (58.27)	4675 (59.33)	815 (51.59)			5248 (58.50)	242 (53.39)		
≤ 6 h	3078 (28.39)	2447 (26.81)	631 (38.36)			2871 (28.03)	207 (36.20)		
≥ 9 h	1503 (13.34)	1318 (13.86)	185 (10.04)			1439 (13.47)	64 (10.40)		
ePWV (m/s), Mean (S.E)	8.31 (0.04)	8.14 (0.03)	9.35 (0.08)	t = -14.94	< 0.001	8.26 (0.03)	9.32 (0.13)	t = -8.19	< 0.001
ePWV (m/s), n (%)				$\chi^2 = 217.44$	< 0.001			$\chi^2 = 63.79$	< 0.001
≤ 6.68	2457 (25.00)	2235 (26.51)	222 (15.44)			2375 (25.45)	82 (15.28)		
6.68-7.85	2127 (24.99)	1912 (25.97)	215 (18.81)			2057 (25.20)	70 (20.37)		
7.85-9.49	2313 (25.01)	2003 (25.43)	310 (22.36)			2225 (25.19)	88 (21.28)		
> 9.49	3174 (25.00)	2290 (22.09)	884 (43.39)			2901 (24.16)	273 (43.07)		
CVD, n (%)				$\chi^2 = 121.85$	< 0.001			$\chi^2 = 24.22$	< 0.001
No	7687 (79.76)	6702 (81.86)	985 (66.52)			7387 (80.38)	300 (66.44)		
Yes	2384 (20.24)	1738 (18.14)	646 (33.48)			2171 (19.62)	213 (33.56)		
Diabetes, n (%)				$\chi^2 = 45.03$	< 0.001			$\chi^2 = 9.74$	0.002
No	7836 (82.80)	6709 (83.94)	1127 (75.61)			7484 (83.11)	352 (76.17)		

Yes	2235 (17.20)	1731 (16.06)	504 (24.39)			2074 (16.89)	161 (23.83)		
Hypertension, n (%)				χ^2 = 86.98	< 0.001			χ^2 = 26.59	< 0.001
No	4961 (53.09)	4409 (55.37)	552 (38.69)			4787 (53.77)	174 (38.64)		
Yes	5110 (46.91)	4031 (44.63)	1079 (61.31)			4771 (46.23)	339 (61.36)		
Dyslipidemia, n (%)				χ^2 = 10.42	0.001			χ^2 = 15.68	< 0.001
No	2770 (28.15)	2396 (28.84)	374 (23.83)			2667 (28.53)	103 (20.03)		
Yes	7301 (71.85)	6044 (71.16)	1257 (76.17)			6891 (71.47)	410 (79.97)		
COPD, n (%)				χ^2 = 1042.08	< 0.001			χ^2 = 189.06	< 0.001
No	4852 (50.58)	4724 (57.46)	128 (7.20)			4820 (52.67)	32 (5.80)		
Yes	269 (2.65)	229 (2.72)	40 (2.20)			262 (2.69)	7 (1.77)		
Unknown	4950 (46.77)	3487 (39.83)	1463 (90.60)			4476 (44.64)	474 (92.44)		
Depression, n (%)				χ^2 = 4.95	0.026			χ^2 = 0.48	0.490
No	8108 (79.14)	6847 (79.57)	1261 (76.49)			7709 (79.21)	399 (77.73)		
Yes	1963 (20.86)	1593 (20.43)	370 (23.51)			1849 (20.79)	114 (22.27)		
Cancer, n (%)				χ^2 = 35.33	< 0.001			χ^2 = 13.12	< 0.001
No	9124 (89.77)	7764 (90.65)	1360 (84.20)			8689 (90.05)	435 (83.64)		
Yes	947 (10.23)	676 (9.35)	271 (15.80)			869 (9.95)	78 (16.36)		
BMI, kg/m^2, Mean (S.E)	30.65 (0.11)	30.78 (0.12)	29.82 (0.20)	t = 4.36	< 0.001	30.67 (0.12)	30.15 (0.40)	t = 1.29	0.203
BMI, n (%)				χ^2 = 10.61	0.005			χ^2 = 2.06	0.357
< 25	2079 (20.52)	1684 (19.99)	395 (23.87)			1976 (20.51)	103 (20.74)		
25-30	3255 (32.24)	2687 (32.01)	568 (33.70)			3058 (32.07)	197 (35.96)		
≥ 30	4737 (47.24)	4069 (48.00)	668 (42.43)			4524 (47.42)	213 (43.30)		
WBC, 1000 cells/uL, Mean (S.E)	7.53 (0.04)	7.52 (0.04)	7.62 (0.08)	t = -1.19	0.238	7.53 (0.04)	7.54 (0.12)	t = -0.08	0.937
AST/ALT ratio, Mean (S.E)	1.06 (0.00)	1.05 (0.01)	1.14 (0.01)	t = -7.64	< 0.001	1.06 (0.00)	1.11 (0.02)	t = -2.46	0.017

Creatinine, mg/dL, Mean (S.E)	0.90 (0.00)	0.89 (0.00)	1.00 (0.01)	t = -8.05	< 0.001	0.90 (0.00)	0.99 (0.02)	t = -5.50	< 0.001
Uric acid, mg/dL, Mean (S.E)	5.60 (0.02)	5.58 (0.02)	5.73 (0.05)	t = -2.83	0.006	5.59 (0.02)	5.81 (0.07)	t = -2.89	0.005
Anti-hypertensive agents, n (%)				χ^2 = 84.03	< 0.001			χ^2 = 23.47	< 0.001
No	6066 (64.42)	5338 (66.57)	728 (50.89)			5841 (65.08)	225 (50.35)		
Yes	4005 (35.58)	3102 (33.43)	903 (49.11)			3717 (34.92)	288 (49.65)		
Barbiturates and benzodiazepines, n (%)				χ^2 = 2.59	0.108			χ^2 = 0.32	0.571
No	9639 (95.33)	8103 (95.47)	1536 (94.48)			9158 (95.37)	481 (94.61)		
Yes	432 (4.67)	337 (4.53)	95 (5.52)			400 (4.63)	32 (5.39)		
Try to lose weight, n (%)				χ^2 = 43.09	< 0.001			χ^2 = 6.05	0.049
No	5229 (49.57)	4216 (48.05)	1013 (59.20)			4928 (49.34)	301 (54.55)		
Yes	3481 (36.94)	3056 (37.99)	425 (30.29)			3327 (36.98)	154 (35.96)		
Unknown	1361 (13.49)	1168 (13.96)	193 (10.51)			1303 (13.67)	58 (9.49)		
Follow-up time, months, Mean (S.E)	102.10 (2.34)	100.15 (2.53)	114.40 (3.12)	t = -3.96	< 0.001	101.66 (2.44)	111.59 (4.93)	t = -1.79	0.079

Abbreviation: ALT: Alanine aminotransferase; AST: Aspartate aminotransferase; BMI: Body mass index; COPD: Chronic obstructive pulmonary disease; CVD: Cardiovascular disease; ePWV: Estimated pulse wave velocity; Pir: Poverty-to-income ratio; S.E: Standard error; WBC: White blood cell. Note: t indicates t-test; χ2, chi-square test.
