# Supplementary figures and images for: Association of estimated pulse wave velocity with all-cause mortality and cardiovascular mortality in obstructive sleep apnea patients: results from NHANES
Source: Front Cardiovasc Med. 2025 Jun 12;12:1571610. doi: 10.3389/fcvm.2025.1571610 (PMC12198205; doi:10.3389/fcvm.2025.1571610)

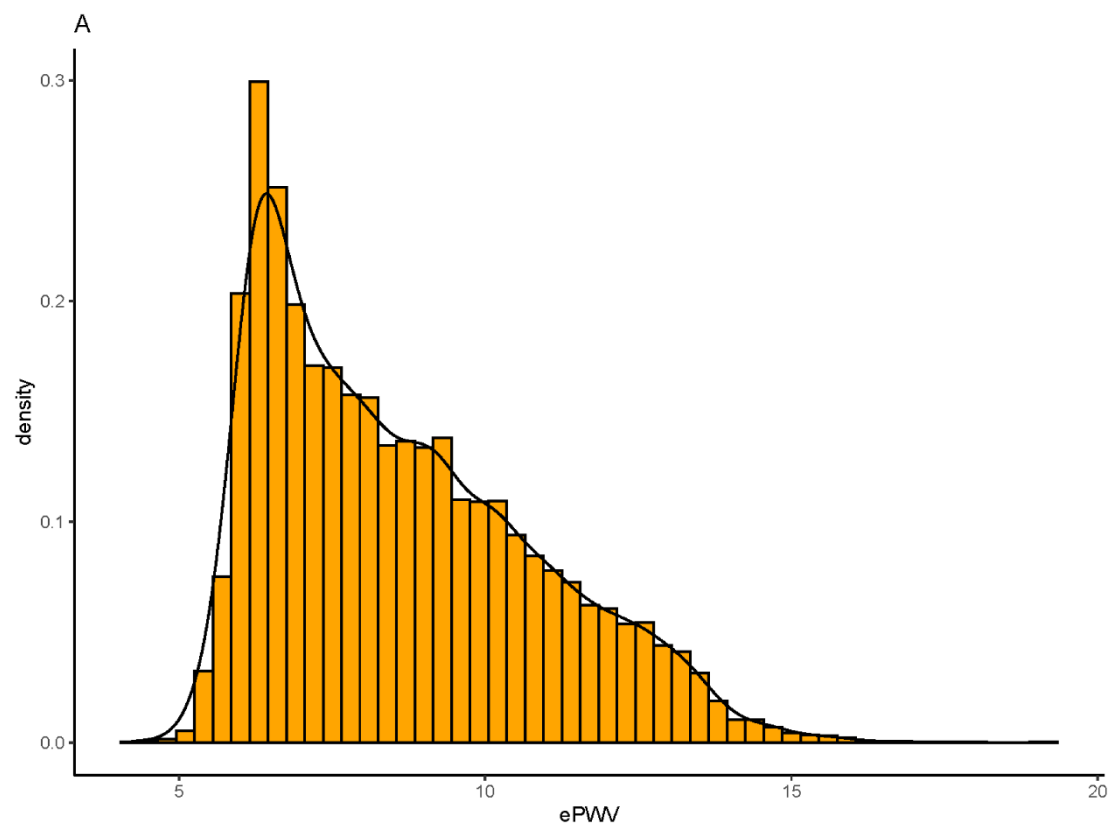

**Figure S1 The distribution of ePWV among OSA patients.**

Supplement: Supplementary file 2 [file Image1.pdf]
